# Seneca Valley Virus Exploits TEM8, a Collagen Receptor Implicated in Tumor Growth

**DOI:** 10.3389/fonc.2018.00506

**Published:** 2018-11-06

**Authors:** David J. Evans, Alexa M. Wasinger, Robert N. Brey, James M. Dunleavey, Brad St. Croix, James G. Bann

**Affiliations:** ^1^Department of Chemistry, Wichita State University, Wichita, KS, United States; ^2^Kinesis Vaccines, LLC, Grayslake, IL, United States; ^3^Tumor Angiogenesis Unit, National Cancer Institute (NCI), National Institutes of Health (NIH), Frederick, MD, United States

**Keywords:** anthrax, Protective Antigen, Seneca Valley Virus, type VI collagen, small cell lung cancer, TEM8/ANTXR1, neuroendocrine tumors

## Abstract

Recent studies reveal that Seneca Valley Virus (SVV) exploits tumor endothelial marker 8 (TEM8) for cellular entry, the same surface receptor pirated by bacterial-derived anthrax toxin. This observation is particularly significant as SVV is a known oncolytic virus which selectively infects and kills tumor cells, particularly those of neuroendocrine origin. TEM8 is a transmembrane glycoprotein that is preferentially upregulated in some tumor cell and tumor-associated stromal cell populations. Both TEM8 and SVV have been evaluated for targeting of tumors of multiple origins, but the connection between the two was previously unknown. Here, we review currently understood interactions between TEM8 and SVV, anthrax protective antigen (PA), and collagen VI, a native binding partner of TEM8, with an emphasis on potential therapeutic directions moving forward.

## Introduction

A recurring hallmark in the effort to develop novel cancer treatments has been identification of biologically derived agents with anti-neoplastic activity. Natural products, including taxanes derived from yew trees and doxorubicin from soil bacteria, remain some of the most effective agents against cancers of multiple origins. More recent work has focused on discovery and characterization of viruses that selectively target tumor cells—i.e., oncolytic viruses. In 2015 the first oncolytic virus, Talimogene Laherparepvec (T-VEC), a genetically modified herpesvirus, was approved for melanoma treatment (1). Fueled by this success, new oncolytic viruses were developed for tumor-specific killing, including polioviruses targeting CD155 in brain cancers (2) and adenoviruses armed with EGFR-targeting, bispecific T-cell engagers (3). Oncolytic viruses can also be engineered to stimulate antigen presentation and improve immunogenic recognition of tumors (1, 4). While oncolytic viruses are being evaluated against many tumor types, better understanding of the mechanisms regulating tumor-selective tropism is needed.

### Identification of TEM8, a novel tumor marker

Proteins selectively expressed either in tumor cells themselves or in surrounding stroma, have contributed greatly to our understanding of cancer progression and uncovered many potential targets for novel cancer therapeutics. One such target, discovered in a transcriptomic screen of human tumor stromal cells (5) is a single-pass cell surface protein called Tumor Endothelial Marker 8 (TEM8). Shortly after its identification TEM8 was found to be a cellular receptor for *Bacillus anthracis*-derived anthrax toxin, and more recently as the cellular receptor for Seneca Valley Virus (SVV) (6, 7). Of note, TEM8 has been shown to bind to the C5 domain of collagen type VI (8). Upregulation of TEM8 seems to promote tumor growth and progression, and multiple therapies targeting TEM8 have shown anti-tumor efficacy in preclinical models (8–11). The recent finding of SVV-TEM8 binding is of special significance as SVV was initially characterized as an oncolytic virus that selectively kills tumor cells, does not replicate in or kill normal human cells, and has a favorable safety profile in clinical trials (12–15). While TEM8 was not evaluated during SVV's therapeutic characterization, its identification as an obligatory cellular receptor suggests patient TEM8 status could be used to stratify patients for SVV therapy in future clinical trials. TEM8 itself has been previously proposed as a target for cancer therapy using monoclonal antibodies, drug-conjugated antibodies, and vaccines (9, 11, 16, 17). TEM8 may also function as a biomarker for different tumors types including, among others, lung, breast, and colorectal cancer (9, 11, 18–20).

### TEM8 as an anthrax toxin receptor and receptor for SVV

While TEM8 was identified as the receptor for anthrax toxin and was alternatively named anthrax toxin receptor-1 (ANTXR1) (7), subsequent studies found a closely related paralogue, capillary morphogenesis protein 2 (CMG2/ANTXR2), that has higher affinity for anthrax toxin and is more widely expressed (21). Both CMG2 or TEM8 can bind anthrax toxin to trigger the first phase of *B. anthracis* pathogenesis (7, 21), but while CMG2 knockout mice are resistant to anthrax toxin challenge, TEM8 knockout mice are not (22). Unlike anthrax toxin, presence of TEM8, but not CMG2, on cells is a necessary prerequisite for binding by SVV (6). Subversion of mammalian receptors is a common tactic for onset of uptake by viruses and bacterial toxins. However, TEM8 is unique as a receptor involved in the pathogenicity of both a bacteria and a virus that infects mammals.

This review aims to provide a backdrop for ongoing research devoted to understanding TEM8 and the interplay between TEM8 and collagen in cancer, and how two unrelated foreign biologics (anthrax toxin and SVV) happen to target the same protein. Additionally, recent findings suggest the potential value of revisiting SVV as an anti-cancer agent, as TEM8 status may inform a therapeutic window for more rational treatment design.

## TEM8 and CMG2 as anthrax toxin receptors

**Figure 1:**
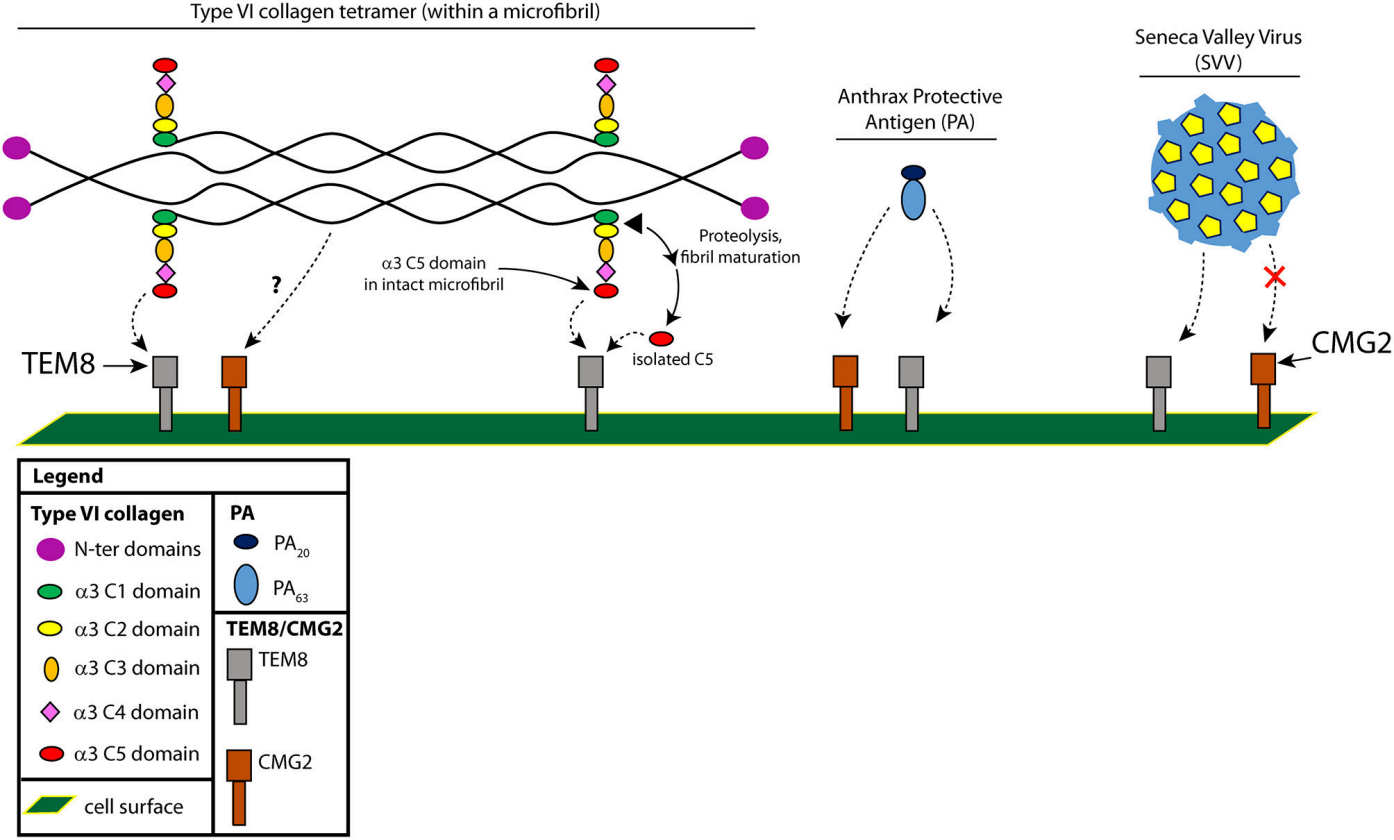
Interactions between TEM8 and type VI collagen, Protective Antigen (PA) and (SVV). The two cell surface receptors, TEM8 and CMG2, can both bind type VI collagen and PA, but only TEM8 can bind SVV. Shown is a type VI collagen tetramer, with each chain comprising three separate α chains [α1(VI), α2(VI), and α3(VI)]. We've highlighted here the C-terminal portion of the α3(VI) chain (C1-C5). The C2-C5 chains are not found in mature fibrils and are proteolytically cleaved by an unknown protease during microfibril maturation; whether C5 binds in the context of a microfibril or in the context of a cleaved C5 domain is not known, so we present both possibilities.

Anthrax toxin consists of three proteins: protective antigen (PA), lethal factor (LF), and edema factor (EF). PA is an 83 kDa protein comprised of four domains, the last of which (domain 4) is responsible for mediating binding to either TEM8 or CMG2 on cells. Following binding, PA domain 1 is cleaved by a membrane-associated furin-class protease to produce a 63 kDa form of PA (Figure 1), which subsequently oligomerizes to form either a heptameric or octameric pre-pore via homophilic binding of domain 3 (23, 24).

TEM8, a ~85 kDa cell surface transmembrane glycoprotein, was originally identified based on its elevated expression in colorectal tumor endothelium (5). Subsequently, TEM8 was found to be elevated in other tumor-associated cell types, including cancer-associated fibroblasts, pericytes and occasionally tumor cells themselves (5, 9, 11, 18, 25). Although TEM8 was the first identified PA receptor, a second cellular receptor, CMG2, was discovered shortly thereafter in endothelial cells, and shares a similar structure to TEM8 (21, 26, 27). TEM8 is highly conserved, with the full-length mouse and human mature proteins sharing 98% amino acid identity (28). Both TEM8 and CMG2 contain an extracellular von Willebrand Factor A (vWA) domain with a metal-ion-dependent adhesion site (MIDAS) which binds PA domain 4 (29). Although the vWA domains of both receptors share 60% homology, CMG2 was found to be the primary receptor responsible for mediating anthrax toxin toxicity (22, 30, 31). Additionally (as mentioned above) CMG2 knockout mice tolerate anthrax toxin challenge, while TEM8 knockout mice do not (32).

## Physiological roles of TEM8 and CMG2

The native physiological function of both anthrax toxin receptors (TEM8 and CMG2) *in vivo* remains largely unknown. The extracellular domains of both proteins share homology with integrins, and interactions with collagen IV, collagen VI and laminin have been demonstrated with CMG2, suggesting a possible role in basement membrane assembly and angiogenesis (27, 33). In human disease, CMG2 mutations have been implicated in hyaline fibromatosis syndrome, a condition characterized by extracellular matrix dysregulation and connective tissue defects due to accumulation of collagen VI. CMG2 was shown to regulate uptake and degradation of collagen type VI through endocytosis (33). Interestingly, this same study found that genetic deletion of collagen VI was sufficient to rescue the major extracellular matrix (ECM) defects found in CMG2 knockout mice. GAPO syndrome, caused by TEM8 inactivating mutations, is a different disease which also involves excess buildup of ECM in various tissues (34). TEM8 knockout mice display many of the same phenotypes found in GAPO patients, including excess ECM deposition, stunted growth and dental abnormalities (10). How TEM8 mediates these phenotypes *in vivo* is currently unclear, although interactions between TEM8 and the ECM are likely involved. Importantly, while TEM8 was discovered to be strongly upregulated in tumor-associated stromal cells, TEM8 knockout mice display normal developmental (retinal angiogenesis) and non-tumor vascular development (wound healing) (10). Furthermore, cell-line derived tumors showed marked growth retardation in TEM8 knockout vs. wildtype mice indicating that TEM8 function in tumor stroma promotes tumor growth (9, 10). Further work exploring the interplay of TEM8 and the ECM *in vivo* should shed light on the pathogenesis of GAPO syndrome and the biology of this currently orphaned receptor.

## Targeting TEM8 in cancer

TEM8 represents an attractive target for novel cancer therapeutics based on its selective upregulation on the cell surface of tumor-associated endothelial cells, pericytes, and cancer-associated fibroblasts (11). TEM8 has been found to be consistently overexpressed in the stroma of many different cancer types (9–11, 35). Notably, TEM8 protein expression patterns are similar to that of collagen VI α3 chain and endotrophin (8, 9, 35–38). Compared to TEM8 wildtype mice, tumor growth in TEM8 knockout mice was markedly reduced for melanoma, lung, breast, and colon cancer models (9, 10). TEM8 becomes highly expressed in human microvascular endothelial cells *in vitro* in response to serum starvation, suggesting that TEM8 may be part of a compensatory pathogenic-related angiogenic pathway (9, 19, 39). TEM8 is overexpressed in the tumor-associated stroma of most solid tumors examined independent of whether or not TEM8 can be detected in the tumor cells (11).

TEM8 is a potential target for antibody-based therapies and vaccination. In several different murine xenograft tumor models, TEM8 blockade using monoclonal antibodies inhibited tumor growth to an extent similar to that observed in TEM8 knockout mice (9). In mice, TEM8 antibodies slowed tumor growth and prolonged survival through a mechanism that may involve function-blocking activity or antibody-dependent cellular cytotoxicity (9). However, no tumor regressions in response to the monotherapy were observed. Preliminary evaluation of several DNA vaccines and vaccine vectors has also demonstrated that active immunity to TEM8 can modify the growth of tumors in mice (40). In particular, vaccination with a DNA vaccine encoding the extracellular domain of TEM8 and Her2/*neu* resulted in prolonged survival after breast tumor cell challenge (233-VSGA1), while a DNA vaccine encoding TEM8 alone was ineffective (16). Treatment with TEM8-Fc fusion proteins or TEM8 chimeric antigen receptor (CAR) T cells has also shown encouraging anti-tumor activity in preclinical studies (38, 41). More recently, a TEM8 antibody-drug-conjugate utilizing monomethyl auristatin E (MMAE), an anti-mitotic microtubule-disrupting agent, induced significant tumor regression in preclinical studies using immunodeficient mice challenged with various human colon, breast, lung, ovarian, and pancreatic tumor xenografts (11). Depending on the model, 10–80% of the mice were tumor free following treatment. Notably, the anti-tumor activity depended upon bystander cleavage of MMAE from the antibody-drug conjugate in stromal cells rather than direct killing of tumor cells. This mechanism allowed for a high intratumoral-localized dose of MMAE through antibody-drug conjugate capture and drug release by stroma (42). Despite the preclinical successes targeting TEM8, a better understanding of its biologic function should aid in the future design of new and improved TEM8-targeted therapeutics going forward.

## Possible interactions of TEM8 with collagen and cleavage products

TEM8 can bind collagen types I and VI and aid in cell spreading and migration on collagen I *in vitro* (8, 43, 44). In addition, *in vitro*, TEM8 has been shown to bind to the C5 domain of the α3 chain of collagen VI (8) (Figure 1). Type VI collagen, which in the monomeric form is comprised predominantly of three chains (α1, α2, and α3), can form dimers and tetramers that are highly cross-linked through disulfide bonds (45). Dimers and tetramers form intracellularly and are deposited on the surface as tetramers. Tetramers then form microfibrils, which require an end-end association of the C-terminal globular domains of the tetramers; maturation into mature fibrils requires further proteolytic processing, likely somewhere between the C1 and C2 domains of collagen α3(VI) (through some undefined protease—Figure 1) (46). The cleaved C5 domain has also been called “endotrophin,” but whether this is solely the C5 domain or includes the other domains (C2-C5) is not known (8, 37).

The interaction between TEM8 and the C5 domain was identified using a yeast two-hybrid screen, and subsequently confirmed by co-immunoprecipitation studies (8). In both lung and esophageal cancers, TEM8 co-localized with the collagen α3(VI) chain. As mentioned above, CMG2 has also been shown to bind type VI collagen, but that collagen was obtained from a commercial vendor (pepsin treated from human placenta). Pepsin digestion yields mainly dimers, tetramers and aggregates of type VI with most of the globular domains (C2-C5) removed (45). Whether CMG2 can bind to type VI through interactions with C5 or whether TEM8 can bind to pepsin-treated type VI is not known.

Although initial studies failed to detect the C5 domain in mature fibrils of type VI collagen (47, 48), in experiments by Lamande et al. who transfected human osteosarcoma SaOS-2 cells with type VI of varying α3 chain lengths, full-length collagen VI microfibrils (with C5) could be observed in cell culture, while cells lacking this domain failed to form microfibrils (46). The C5 domain was also observed in the pericellular matrix of human cartilage. Therefore, processing of the C-terminal domains and removal of C5 likely occurs at an intermediate phase of fibril assembly (after microfibrils have formed but before full maturation to fibrils), and may involve TEM8 to promote or regulate this processing event.

## Collagen VI and upregulation in cancer

Collagen VI was found to be upregulated in malignant tumors of human breast cancer patients, and antibodies specifically targeting the C-terminal C5 domain suggested a greater abundance of this region in or around tumor cells (49). Endotrophin, which includes the cleaved C5 domain of collagen VI α3, was found to be responsible for the tumor-promoting effects of collagen VI (50). A major source of endotrophin in obesity-related cancers is collagen VI derived from tumor-associated adipocytes, which promotes tissue fibrosis by stimulating additional production of collagen VI and other extracellular matrix molecules, leading ultimately to a pro-inflammatory response (51). The presence of endotrophin in the microenvironment of MMTV-PyMT mammary tumors was found to drive primary tumor growth and pulmonary metastasis through an enhanced expansion of the tumor stroma (50, 51). These effects can be largely suppressed using monoclonal antibodies directed against endotrophin (50). The question now is whether an interaction with TEM8 is part of the mechanism responsible for the tumor-promoting activity of endotrophin, since TEM8 is capable of binding the C5 domain (see above and Figure 1).

## TEM8 binds to SVV and is essential for infection

TEM8 was recently identified as the cellular receptor for Seneca Valley Virus, SVV (6). SVV belongs to the family *Picornavirus*, which is a positive strand RNA virus that appears to be non-pathogenic in humans, although SVV and related viruses cause outbreaks of vesicular disease in swine throughout the world (52). The requirement for TEM8 expression in SVV permissivity was experimentally determined after pooled genome-wide loss-of-function studies in a haploid cell line (6). Further validation was obtained by comparing the permissivity of TEM8 wildtype and knockout clones of the H446 neuroendocrine small cell lung cancer (SCLC) cell line. The exogenous expression of TEM8 in non-permissive H69 and H146 SCLC cells allowed for effective SVV entry and killing. TEM8, but not CMG2 was also shown to directly bind SVV via co-immunoprecipitation studies (6) and cryo-electron microscopy of SVV-TEM8 mixtures revealed a regular labeling of SVV's capsid with TEM8 molecules, confirming its status as the SVV receptor (53).

The finding that a bacterial toxin protein (PA) and a virus (SVV) target the same receptor is surprising but not entirely new. The first account of a potentially shared receptor for a bacterial toxin and a virus is the binding of the *Clostridium difficile* toxin TcdB to the poliovirus receptor-like 3 (PVRL3). PVRL3 is a member of the poliovirus receptor family of nectins that are involved in receptor activity for several viruses including herpes virus, measles and poliovirus (54). The interaction between TEM8 and SVV has also been shown to be specific, as SVV has not been shown to interact with CMG2 (Figure 1).

## SVV and oncolytic viral therapy

SVV has shown effective reduction of established human xenograft tumors grown in Athymic (nu/nu) immunodeficient mice (14). Additional studies in severe combined immunodeficient (SCID) mice with SVV successfully controlled or eradicated xenograft tumors expressing neuroendocrine features (55, 56). While oncolytic viral therapy, on the whole, represents a promising approach to treat a variety of cancers, SVV is particularly interesting as it can be administered intravenously and can rapidly replicate without the need for a DNA intermediate using host machinery.

The discovery of TEM8's role as the cellular receptor for SVV allows for a more focused approach to SVV as a therapeutic. Retrospective analysis revealed that SCLC cell lines that were susceptible to SVV also expressed TEM8 mRNA in the Cancer Cell Line Encyclopedia (CCLE) database (6). Thus, TEM8 expression appears to be highly correlated with susceptibility to SVV replication in SCLC cell lines. However, TEM8 expression alone was not sufficient for permissivity. SCLC cell lines that supported replication of SVV also displayed downregulation or defects in genes related to type I interferon signaling (6). These results suggest that additional factors, particularly those involved with innate antiviral immunity may regulate oncolytic activity of SVV beyond cellular binding and internalization, and another report suggested a role for sialic acid in regulating SVV infection of glioblastoma (57). Earlier studies from Charles Rudin's group also identified transcription factors NEUROD1 and ASCL1 as predictors of SVV replication, where *ASCL1* was highly expressed in non-permissive lines and *NEUROD1* highly expressed in SVV-permissive cells (56).

In two phase 1 clinical trials (13, 15), SVV therapy administered via intravenous infusion was safe but also associated with rapid immune recognition, which most likely muted its anti-tumor effect. A phase II trial showed similar immune responses and was terminated after interim analysis declared futility (12). Although early human trials of SVV therapy for neuroendocrine cancers have not yet demonstrated efficacy, the requirement of TEM8 for SVV effectiveness suggests that TEM8 expression may have diagnostic value for identifying optimal patient populations for future SVV trials.

## Conclusions

The discovery of TEM8 as the receptor for both SVV and anthrax toxin is striking, as two distinct foreign biologic agents are now known to target the same protein receptor. It appears likely that TEM8 plays a similar role for both agents, namely cellular entry. The recent finding that CMG2 binds to type VI collagen and is involved in its turnover suggests that perhaps TEM8 is also involved ECM remodeling. It is possible that SVV and anthrax toxin share a similar internalization mechanism, although the lack of SVV-CMG2 binding may indicate a relatively unique pathway for SVV. Nonetheless, both agents have evolved independent mechanisms to exploit the same receptor for cellular entry. Several questions remain - does TEM8 binding to collagen preclude an interaction with PA or SVV, or does it facilitate an interaction? Is the interaction with type VI limited to the C5 domain? Future studies of SVV/TEM8 interactions should examine the binding site of the viral envelope to TEM8 through mutagenesis assays, particularly in the MIDAS domain, as well as with previously described monoclonal antibodies which block PA binding (9). Despite the current knowledge gap surrounding specificity of SVV for TEM8, lack of CMG2 binding suggests that characterizing the nature of the viral-receptor interaction could open a window for development of new targeted therapies specific for TEM8, but not CMG2. Additionally, it is possible that SVV could be repurposed to deliver genetic cargo to the stroma to synergize with other therapies, including targeted agents like antibody-drug-conjugates or CAR-T cells. TEM8 screening could also help stratify patients and provide a new path forward for improving clinical trials with SVV, and further optimize the therapeutic window for these trials.

## Author contributions

All authors listed have made a substantial, direct and intellectual contribution to the work, and approved it for publication.

### Conflict of interest statement

The authors declare that the research was conducted in the absence of any commercial or financial relationships that could be construed as a potential conflict of interest.

## Abbreviations

CMG2: capillary morphogenesis protein 2
TEM8: tumor endothelial marker 8
PA: protective antigen
SVV: Seneca valley virus
α3(VI): the a3 chain of type VI collagen
SCLC: small-cell lung cancer.
